# Manubrium-limited ministernotomy versus conventional sternotomy for aortic valve replacement (MAVRIC): study protocol for a randomised controlled trial

**DOI:** 10.1186/s13063-016-1768-4

**Published:** 2017-01-28

**Authors:** Enoch Akowuah, Andrew T. Goodwin, W. Andrew Owens, Helen C. Hancock, Rebecca Maier, Adetayo Kasim, Adrian Mellor, Khalid Khan, Gavin Murphy, James Mason

**Affiliations:** 1 Cardiothoracic Division, The James Cook University Hospital, South Tees Hospitals NHS Foundation Trust, Marton Road, Middlesbrough, TS4 3BW UK; 2 Durham Clinical Trials Unit, School of Medicine, Pharmacy and Health, Durham University, Queen’s Campus, University Boulevard, Thornaby, Stockton-on-Tees, TS17 6BH UK; 3 Department of Cardiovascular Sciences, University of Leicester, Clinical Sciences Wing, Glenfield General Hospital, Leicester, LE3 9QP UK; 4 Warwick Medical School, University of Warwick, Coventry, CV4 7AL UK

**Keywords:** Aortic valve replacement (AVR), Manubrium-limited ministernotomy, Minimally invasive aortic valve replacement, Sternotomy, Red blood cell transfusion, Inflammatory response

## Abstract

**Background:**

Aortic valve replacement is one of the most common cardiac surgical procedures performed worldwide. Conventional aortic valve replacement surgery is performed via a median sternotomy; the sternum is divided completely from the sternal notch to the xiphisternum. Minimally invasive aortic valve replacement, using a new technique called manubrium-limited ministernotomy, divides only the manubrium from the sternal notch to 1 cm below the manubrio-sternal junction.

More than one third of patients undergoing conventional sternotomy develop clinically significant bleeding requiring post-operative red blood cell transfusion. Case series data suggest a potentially clinically significant difference in red blood cell transfusion requirements between the two techniques. Given the implications for National Health Service resources and patient outcomes, a definitive trial is needed.

**Methods/design:**

This is a single-centre, single-blind, randomised controlled trial comparing aortic valve replacement surgery using manubrium-limited ministernotomy (intervention) and conventional median sternotomy (usual care). Two hundred and seventy patients will be randomised in a 1:1 ratio between the intervention and control arms, stratified by baseline logistic EuroSCORE and haemoglobin value. Patients will be followed for 12 weeks from discharge following their index operation. The primary outcome is the proportion of patients who receive a red blood cell transfusion post-operatively within 7 days of surgery. Secondary outcomes include red blood cell and blood product transfusions, blood loss, re-operation rates, sternal wound pain, quality of life, markers of inflammatory response, hospital discharge, health care utilisation, cost and cost effectiveness and adverse events.

**Discussion:**

This is the first trial to examine aortic valve replacement via manubrium-limited ministernotomy versus conventional sternotomy when comparing red blood cell transfusion rates following surgery. Surgical trials present significant challenges; strengths of this trial include a rigorous research design, standardised surgery performed by experienced consultant cardiothoracic surgeons, an agreed anaesthetic regimen, patient blinding and consultant-led patient recruitment. The MAVRIC trial will demonstrate that complex surgical trials can be delivered to exemplary standards and provide the community with the knowledge required to inform future care for patients requiring aortic valve replacement surgery.

**Trial registration:**

International Standard Randomised Controlled Trial Number (ISRCTN) ISRCTN29567910. Registered on 3 February 2014.

**Electronic supplementary material:**

The online version of this article (doi:10.1186/s13063-016-1768-4) contains supplementary material, which is available to authorized users.

## Background

### Aortic valve replacement (AVR)

AVR is one of the most common cardiac surgical procedures performed worldwide [1, 2]. Patients suffer symptoms of chest pain, shortness of breath and dizziness as a result of aortic stenosis or regurgitation. Nearly 10,000 patients undergo AVR surgery in the UK every year [2]. Patient outcomes of AVR performed in the UK from 2004 to 2009 [3] showed a 26% increase in the number of patients undergoing surgery during this period. At The James Cook University Hospital (JCUH) in the North of England, an audit over the same time period confirmed a 24% increase in the number of AVR operations. It is anticipated that the number of AVR operations will continue to increase.

### Blood transfusion following AVR

There is significant morbidity associated with AVR surgery. Consequently, blood loss and the subsequent requirement for transfusion of red blood cells (RBCs) and blood products are key indicators of quality. More than one-third of patients undergoing conventional sternotomy develop clinically significant bleeding and require a post-operative RBC transfusion [4, 5]. Blood transfusion can have adverse clinical effects including post-operative lung injury, organ dysfunction, confusion, and immunosuppression [6]; complications of transfusion have been directly linked to prolonged hospital stay and increased mortality after cardiac surgery [5, 7–12]. Additionally, there is a small risk of transmitting viral infection from blood donor to recipient [13]. Currently, cardiac surgical procedures use 6% of all donor blood available in the UK [14]. An analysis of patients over 5 years from the Society for Cardiothoracic Surgery in Great Britain and Ireland National Database indicated that of 41,227 patients who underwent AVR surgery, 2342 (6%) required a second operation due to excessive bleeding [3].

Retrospective studies have shown that blood loss and transfusion requirements are significantly less with minimally invasive AVR [15–17]; however, most reported using a fourth space ministernotomy incision rather than a manubrium-limited approach. No study thus far has tested RBC transfusion requirements in a randomised controlled trial using manubrium-limited ministernotomy.

## Rationale for choice of comparators'

### Surgical techniques in AVR

#### Usual care: conventional sternotomy

Conventional surgery for AVR is performed via a median sternotomy, in which the sternum is divided completely from the sternal notch to the xiphisternum. The operation includes cardiopulmonary bypass established by siting cannulas in the right atrium and ascending aorta. The heart is stopped and the valve is replaced.

#### Intervention under study: minimally invasive ministernotomy

The new technique of manubrium-limited ministernotomy divides only the top quarter of the sternum from the sternal notch to 1 cm below the manubrio-sternal junction; this enables access to perform the AVR. Potential benefits may include reductions in bleeding, post-operative pain, inflammatory response, hospital stay and time away from work. The cardiothoracic surgical community are enthusiastic about the procedure; however, they are clear that definitive benefit needs to be demonstrated in a randomised controlled trial before widespread adoption of the technique.

### Inflammatory markers

In minimally invasive cardiac surgical procedures, there is less tissue trauma and the right atrium is not directly cannulated; conversely, cardiopulmonary bypass and aortic cross-clamp times are longer. The mechanism for any observed benefits of the minimally invasive approach is unconfirmed, but may be due to a difference in systemic inflammatory response (SIR). SIR can be measured by monitoring the profile of cytokines in plasma.

This trial will determine if there is a difference in SIR to AVR via manubrium-limited ministernotomy when compared to conventional sternotomy by measuring inflammatory markers at pre- and post-surgical time points. We will seek to understand the mechanism underlying the observations we make. Our hypothesis is that patients who receive a sternotomy will bleed more and require more blood transfusions. The excess bleeding might be a direct result of the increased surgical trauma or as a result of an increased SIR to sternotomy. A SIR may have wide-ranging post-operative effects and has previously been shown to increase atrial fibrillation and acute kidney injury, impair wound healing and reduce post-operative haemostasis [18].

### Trial rationale

Case series data at The JCUH suggest a potentially significant and clinically important difference in the need for RBC transfusion when comparing patients undergoing conventional and manubrium-limited surgery. Given the implications of transfusion for National Health Service (NHS) resources and patient outcomes, and the potential benefits from this new technique, there is a need for a definitive trial. There has been one trial in the UK evaluating the fourth space median (minimally invasive) sternotomy (PB-PG-0408-16296; ISRCTN 58128724); this trial is now closed to recruitment and is in follow-up. Thus far, no randomised trial has compared manubrium-limited ministernotomy to conventional sternotomy for AVR.

The need for AVR is increasing and, with an ageing population, the balance of surgical risk will become less favourable given the greater level of co-morbidity in older populations. Importantly, this new approach also has the potential to reduce the risk of post-operative lung injury, organ dysfunction and immunosuppression, as well as reduce the burden on already overstretched blood transfusion services. A robust trial of the manubrium-limited technique compared with conventional surgery is imperative and timely to ensure that appropriate surgical strategies deliver improved patient outcomes and efficient use of scarce NHS resources.

The trial will run according to the principles of International Conference on Harmonisation (ICH)-Good Clinical Practice (GCP) and in accordance with relevant UK legislation and the trial protocol.

## Methods/design

### Objectives

This trial will investigate whether new manubrium-limited surgery (intervention) reduces RBC transfusion rates compared to conventional cardiac surgery (control) for patients undergoing aortic valve replacement. The null hypothesis is that there will be no difference in the proportion of patients receiving RBC transfusion after manubrium-limited ministernotomy when compared to conventional sternotomy for AVR.

### Trial design

This is a single-centre, single-blind, randomised controlled superiority trial comparing patients undergoing AVR via manubrium-limited ministernotomy (intervention under study) or conventional median sternotomy (control arm/usual care); randomisation will be performed using random permuted blocks with a 1:1 allocation.

**Fig. 1:**
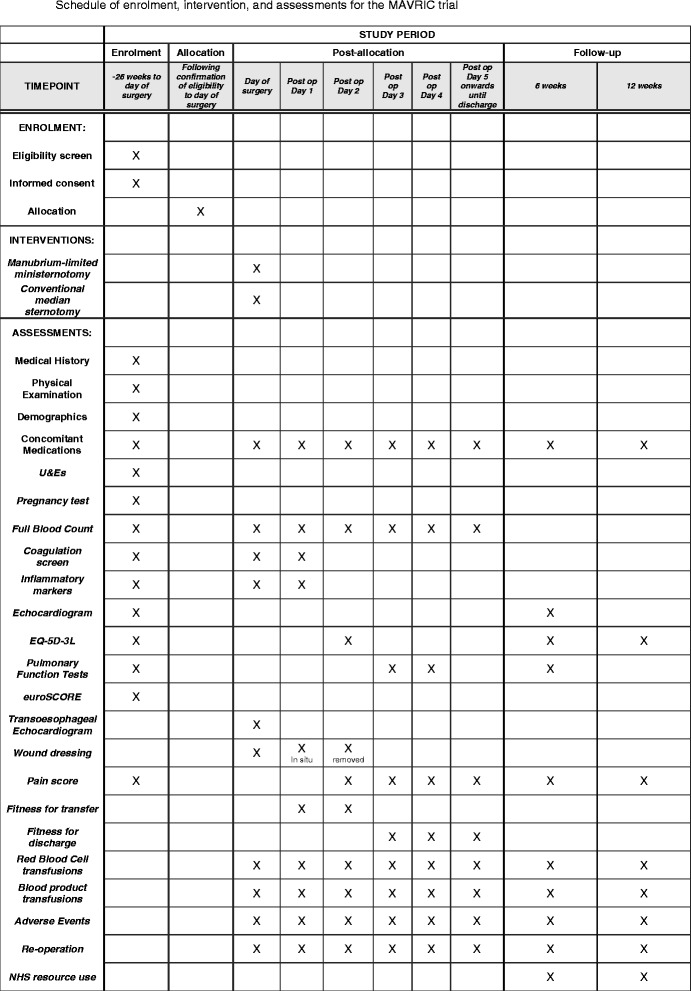
Schedule of enrolment, intervention and assessments for the MAVRIC trial

The manubrium-limited ministernotomy versus conventional sternotomy for aortic valve replacement (MAVRIC) trial protocol was written in accordance with the Standard Protocol Items: Recommendations for Interventional Trials (SPIRIT) checklist (see Additional file 1) [19, 20]. The schedule of this trial is shown in Fig. 1.

### Trial setting

The study aims to recruit 270 patients in a single NHS Trust in the North of England.

### Eligibility criteria

#### Inclusion criteria

Patients are eligible for the study if they:

1. Are aged 18 years or older at the time of consent
2. Require first-time, non-emergency, isolated AVR; surgery
3. Are able; and willing to provide written informed consent.

#### Exclusion criteria

Patients are excluded from entering the study if they:

1. Require concomitant cardiac procedure(s)
2. Have a haemoglobin level <90 g/L
3. Are pregnant; Are unable to stop currently prescribed treatment affecting clotting
4. Have a haematological condition that would affect participation in the trial
5. Have infective endocarditis
6. Are prevented from having RBCs and blood products according to a system of beliefs
7. Have any other medical, psychiatric and or social reason that precludes participation.

#### Eligibility check

Participants have their eligibility checked and confirmed within the 14 days prior to surgery. Eligibility is confirmed by one of the three operating cardiac surgeons who are clinical investigators for this trial.

### Interventions

#### Manubrium-limited ministernotomy (intervention)

**Fig. 2:**
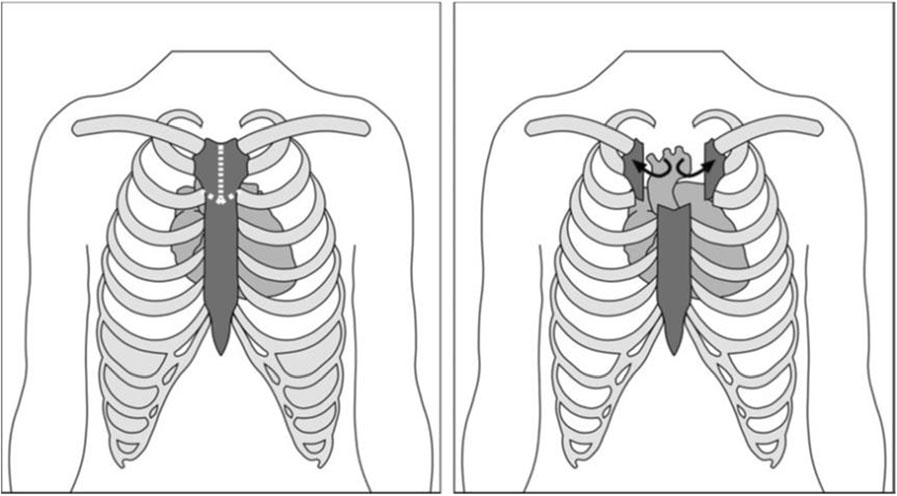
Division and transection of the sternum in a manubrium-limited ministernotomy

Manubrium-limited ministernotomy (intervention arm) is performed using systemic normothermia. An incision is made from the sternal notch to the second intercostal space. The manubrium is divided longitudinally in the midline. The sternum is then transected in both directions from the second intercostal spaces until the midline incision is reached, creating a V shape. This procedure is depicted in Fig. 2. Aortic cannulation is through the ascending aorta. As the right atrium is poorly visualised with this technique, venous cannulation is percutaneous through the femoral vein (using a Seldinger technique guided by transoesophageal echocardiography). Vacuum assist is used as necessary to aid venous drainage. Antegrade cardioplegia is used for myocardial protection, and venting is via the pulmonary artery. A transverse aortotomy is performed, followed by standard aortic valve insertion using interrupted nonpledgeted braided sutures. The aortotomy is closed in a single or double layer. One pericardial drain and ventricular pacing wires are placed in all patients. Atrial wires are placed if needed. These steps are performed prior to removing the cross-clamp to facilitate the view of the right atrium and ventricle. The sternum is closed with two wires in the manubrium and two wires from the body of the sternum up to the manubrium.

#### Conventional median sternotomy (control)

For the conventional technique, a standard median sternotomy is performed using systemic normothermia. Cannulation is via the ascending aorta with two-stage right atrial cannulation for venous drainage. Venting of the left ventricle is achieved via the pulmonary artery, and myocardial protection is with cold blood antegrade cardioplegia. All valves are inserted using interrupted sutures.

During the trial, both operations are performed in accordance with an agreed and standardised anaesthetic protocol. Patients are given lorazepam as a pre-medication, followed by anaesthesia with propofol, fentanyl, rocuronium bromide and morphine. All patients are given a total dose of tranexamic acid (TXA) at 30 mg/kg. Where patients have a pre-surgical creatinine >200 mmol/L, the dose of TXA is halved to 15 mg/kg. Prior to cardiopulmonary bypass, systemic anticoagulation is achieved with heparin given at a dose that achieves an activated clotting time (ACT) of greater than 400 seconds. Fresh frozen plasma (FFP) is administered if the target ACT is not reached. During cardiopulmonary bypass, haemoglobin (Hb) is kept at 60 g/L or above. Haemofiltration followed by RBC transfusion may be required to achieve this. Following cardiopulmonary bypass (CPB), protamine will be administered to reverse heparin, according to the dose of heparin given. Blood products may be used intra-operatively in the presence of excessive blood loss. RBC salvage will be used in all patients.

All patients have the new aortic valve assessed at the end of surgery using a transoesophageal echocardiogram (TOE). Details of this, as well as any additional surgical intervention, including conversion to conventional sternotomy from manubrium-limited sternotomy, and any further TOE are recorded.

#### Post-operative warfarin and aspirin administration

Post-operatively, all patients having a biological prosthesis begin 75 mg aspirin on the morning of the day following surgery. All patients having a mechanical prosthesis commence on warfarin on the evening of the day following surgery.

#### Post-operative assessments and procedures

The post-operative period (and trial protocol in relation to RBC and other blood product transfusions) begins once the patient has been admitted to the Cardiac Intensive Care Unit (CICU). Residual blood after cardiopulmonary bypass that has been bagged may all be given as a transfusion intravenously; the transfusion of this residual blood is commenced prior to CICU admission.

### Blood and blood product usage following surgery

The post-operative RBC transfusion and blood product transfusion processes for this trial begin from the point of admission to the CICU. All residual blood from the CPB reservoir and cell salvaged blood is returned to the patient; the following transfusion processes are implemented following complete transfusion of this blood and continue until a patient is discharged following their index operation.

Trial patients receive a RBC transfusion if at least one of the following criteria is met:

- Their Hb is <80 g/L.
- A diagnosis of post-operative bleeding is made as defined by ≥400 ml/h blood loss or ≥100 ml/h for ≥ 4 h with Hb ≥80 g/L.
- Blood loss leading to haemodynamic instability occurs irrespective of thromboelastography (TEG) and clotting profile results.

Trial patients receive a blood product transfusion if both of the following criteria are met:

- A diagnosis of post-operative bleeding occurs as defined by ≥400 ml/h blood loss or ≥100 ml/h for ≥ 4 h.
- TEG or coagulation guided transfusion is indicated.

Clinicians are able to transfuse, or decide not to transfuse, in violation of the protocol parameters; their reason for doing so will be recorded.

### Outcomes

#### Primary outcome

The primary outcome is the proportion of patients who receive a RBC transfusion post-operatively and within 7 days of AVR surgery.

#### Secondary outcomes

Secondary outcomes are:

- The proportion of patients who receive a RBC transfusion during the intra-operative period and the entire post-operative hospital stay
- The mean number of RBC units transfused during the intra-operative period, post-operative period (within the 7 days following AVR surgery) and the entire post-operative hospital stay
- The proportion of patients receiving blood product transfusions during the intra-operative period, within the 7 days following AVR surgery and during the entire hospital stay
- The mean number of blood product transfusions received during the intra-operative period, within the 7 days following AVR surgery and during the entire hospital stay
- Mean post-operative blood loss (millilitres) measured from chest drains at 6 and 12 hours, and at the time of drain removal, following AVR surgery
- Operative success as defined by transthoracic echocardiographic assessment of left ventricular function, and degree of aortic regurgitation, within 6 weeks of AVR surgery
- Mean post-operative changes in haemoglobin (Hb) within the index hospital stay
- Mean post-operative changes in inflammatory markers on admission to CICU and on day 1 following AVR surgery
- Proportion of patients reporting moderate or severe post-operative sternal wound pain, measured daily using an 11-point numerical rating scale developed by the trial team, until patient is fit for hospital discharge, and at 6 and 12 weeks following AVR surgery
- Rates of re-operation following index AVR surgery until 12 weeks
- Rate of conversion to conventional AVR during index surgery
- Changes in forced expiratory volume and forced vital capacity on days 3 and 4 and at 6 weeks following AVR surgery
- EuroQoL EQ-5D-3L [21] scores, captured at baseline and on day 2, and 6 weeks and 12 weeks following AVR surgery, will be converted to health status scores using the value set (time trade-off) [22] and provide patient-level quality-adjusted life year (QALY) estimates as a health outcome [23]. The EQ-5D-3L is a validated, self-reported outcome measure consisting of five dimensions: mobility, self-care, usual activity, pain/discomfort, anxiety/depression. Each dimension has three levels of response.
- Mean time at which patients are fit for discharge from hospital following AVR surgery
- Health care utilisation during hospital stay and following discharge to 12 weeks, from medical note review, GP records and patient reports
- Cost and cost effectiveness estimated from QALY estimates and health care utilisation valued using national reference costs to 12 weeks
- Rates of related adverse events during the 12 weeks following surgery including severity.

### Participant timeline

**Fig. 3:**
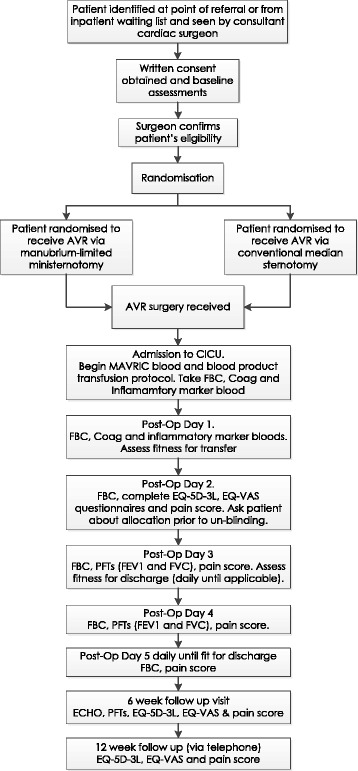
Flow chart of the patient pathway for the MAVRIC trial

Patients are followed for 12 weeks, with follow-up at 6 weeks and 12 weeks after discharge from hospital following their index AVR operation. Figure 3 provides a flow chart of the patient pathway in the MAVRIC trial.

### Sample size calculation

This trial will determine if manubrium-limited ministernotomy is an appropriate clinical alternative to the existing operation (conventional sternotomy) in terms of RBC transfusion requirements in the 7 days following index surgery. Currently, there is clinical and policy equipoise with no intention to extend the use of the new procedure until high-quality randomised controlled trial evidence is available.

Using Fisher’s exact test, 90% power, 5% alpha, 260 patients are required to detect a 17% reduction in the proportion of patients requiring RBC transfusion (13% compared with 30%), using a two-sided test. Recruitment will continue until the target sample size is reached and 260 patients are contributing to the primary outcome.

### Recruitment

Patients undergoing isolated AVR surgery will be identified at the point of referral or from the inpatient waiting list by the clinical team, and will be approached by a member of the research team about participation in MAVRIC. Patients will be consented by a Consultant Cardiothoracic Surgeon or a Surgical Registrar.

### Allocation

Following consent, eligible patients are randomised to receive AVR by manubrium-limited ministernotomy or by conventional sternotomy following confirmation of eligibility. Randomisation is made using a permuted block randomisation, stratified by logistic EuroSCORE [24] (low risk 0–3.50%, moderate risk 3.51–7.5% and high risk >7.5%) and by pre-operative Hb (90–125 g/L, 126–140 g/L, >140 g/L). A web-based randomisation system, managed by Durham Clinical Trials Unit (DCTU), will ensure concealment of allocation.

### Blinding

This is a single-blind trial. Patients are not informed of the type of sternotomy they are planned to receive, or do receive, until completion of the pain assessment on day 2 following their operation. To enable blinding post-operatively, all patients have a trial-specific opaque dressing applied to their sternal wound and to their groin.

#### Measures taken to avoid bias

This trial incorporates a number of methods to avoid bias:

- Concealment of allocation will be achieved through a web-based randomisation system, described above, managed by DCTU. Named clinical research team members enter a minimum data set per patient before individual allocation to type of sternotomy is provided.
- Three consultant cardiothoracic surgeons perform all operations as part of this trial. Each is expert in both techniques and does not delegate to other trainee or consultant surgeons.
- Criteria for blood and blood product transfusions are detailed in the protocol and followed for all patients. Clinical staff members in all cardiothoracic wards follow this protocol. Where clinical need requires blood to be given outside of the protocol, this is documented and described. The trigger for all transfusions is recorded.
- Patients are blind to the sternotomy procedure, both planned and received, for 2 days following their index surgery. All have an opaque dressing applied to both the sternum and the groin to facilitate blinding; these are only removed, and the patient informed, following their day 2 trial assessments unless clinical need requires earlier removal. Sternal wound pain is assessed using an 11-point numerical rating scale, with all analgesic medication taken in the preceding 4 hours recorded.
- Fitness for discharge is measured using defined physiotherapy and clinical criteria; these are assessed daily from day 3 by a research team physiotherapist and by the surgical research team. The date that both physiotherapy and clinical criteria are met is defined as the date the patient is fit for discharge. The date of actual discharge is also recorded.
- Where patients choose to withdraw from the study prior to 7 days following their index surgery, permission is sought to continue data collection to support analysis of the primary endpoint.

### Data collection methods

#### Baseline assessments

In addition to usual care procedures, baseline assessments take place following consent and prior to surgery.

#### Cardiovascular and significant current and past medical history

A full medical history is recorded for each patient at baseline and includes details of all clinically significant past medical conditions and all clinically significant on-going medical conditions including full cardiovascular history.

#### Physical assessment

A physical assessment of height (measured in centimetres) and weight (measured in kilograms) determines body mass index.

#### Current medications

A full list of the generic names of relevant medications taken by the patient is recorded within 14 days before surgery. The information includes frequency and dose. Changes or additions are recorded from baseline until the 12-week follow-up visit.

For patients in both trial arms, pre-operative antiplatelet drugs (including clopidogrel and aspirin) and anticoagulants (including heparin and warfarin) are discontinued 5 days prior to surgery. The exception is aspirin, which is stopped 5 days prior to surgery where possible; however, continuation until the day of surgery does not exclude a patient from the trial, and is recorded. These drugs may be re-started following surgery at the discretion of the clinical team. Dates for re-starting medications are recorded.

#### International normalized ratio (INR) checks for patients taking warfarin

Patients on warfarin have their INR checked as part of routine care on admission to hospital for their index surgery. Where an INR is ≤1.5, the patient proceeds to surgery. Where a patient’s INR is >1.5, appropriate treatment may be given and surgery may need to be delayed. The INR for patients taking warfarin must be ≤1.5 prior to surgery.

#### Demographic information

The following demographic data are recorded:

- Age
- Gender
- Ethnicity.

#### Blood tests

Blood tests are taken within 14 days prior to surgery and prior to randomisation:

- Urea and electrolytes (sodium, potassium, creatinine, urea)
- Pregnancy test
- Full blood count (haemoglobin, haematocrit, platelets, white cell count)
- Coagulation screen (prothrombin time (PT), prothrombin time ratio (PTR), activated partial thromboplastin time (APTT), activated partial thromboplastin time ratio (APTTR)
- Inflammatory markers.

Out of normal range blood parameters are assessed by the clinical team to confirm that there are no clinically significant findings that would affect continuation in the trial. The value of haemoglobin taken up to 14 days pre-surgery is used as a stratifying variable for randomisation.

Patients also have blood samples (stored as plasma) taken pre-operatively, on admission to CICU and 24 h post-operatively. These are analysed to explore the following null hypotheses:

1. That there will be no difference between peri-operative inflammatory markers (IL-6, IL-8, IL-10) and markers of endothelial inflammation (ICAM-1 or CD62E) between those undergoing AVR via manubrium-limited ministernotomy when compared to AVR via conventional sternotomy
2. That there is no correlation between the number and proportion of patients who receive a RBC transfusion and the number of units transfused and peri-operative inflammatory markers (IL-6, IL-8, IL-10).

#### Echocardiogram

Results from the latest echocardiogram (echo) pre-surgery are recorded. If an echo has not been done within 39 weeks (9 months) of consent, this is repeated at baseline.

#### Pulmonary function tests

Pulmonary function tests of forced expiratory volume (FEV1) and forced vital capacity (FVC) are performed at baseline with patients sitting for both assessments. These assessments are repeated on days 3 and 4, and at 6 weeks following discharge from hospital after their index surgery.

#### EuroSCORE

Logistic EuroSCORE [24] is determined prior to randomisation to be used as a stratifying variable, with the score recorded. The elements that determined the logistic EuroSCORE pre-operatively are also recorded. EuroSCORE II [25] and the elements that determine this score are also recorded.

#### Quality of life assessment (EuroQoL EQ-5D-3L [22])

Each patient completes the EuroQoL EQ-5D-3L [21] questionnaires at baseline. If the patient is physically unable to complete the questionnaires, or the assessment is being performed over the telephone, the research team will administer them to the patient, who dictates their answers. The details of who is recording the patient’s responses are noted. Questionnaires are repeated at day 2, 6 weeks and 12 weeks (3 months) following discharge from hospital.

#### Assessment of pain

Pain is assessed within 14 days prior to index surgery using an 11-point numerical rating scale. Pain is also assessed post-operatively (daily from post-operative day 2 until the patient is deemed ‘fit for discharge’), and at follow-up (6 and 12 weeks following discharge).

### Retention of participants

Patients who withdraw have all data collected up until the point of withdrawal included in the study except where withdrawal is due to a related adverse event (AE), in which case the patient is followed until a stable outcome is achieved.

### Data management

The study is managed by the Chief Investigator with support from DCTU.

Study data are recorded in each patient’s medical notes before being entered onto electronic Case Report Forms (e-CRFs). Data entered into the e-CRF must be consistent with the information in the medical notes. Discrepancies are noted and explained. Un-anonymised data are held on site in accordance with local Trust policies. Patients are identified by a unique study number at enrolment. All data passed to DCTU have patient identifiers removed, except date of birth, gender, ethnicity and unique study ID. All data are handled in a confidential manner by DCTU, the research team and by members of the Data Monitoring Committee (DMC) and Trial Steering Committee (TSC).

### Statistical methods

The null hypothesis is that there will be no difference in the proportion of patients receiving RBC transfusion after manubrium-limited ministernotomy when compared to conventional sternotomy for AVR.

This trial will determine if manubrium-limited ministernotomy is an appropriate clinical alternative to the existing operation (conventional sternotomy) in terms of RBC transfusion requirements in the 7 days following index surgery. An analysis of the primary endpoint will be conducted using Fisher’s exact test. A sensitivity analysis will also be performed for the primary endpoint using a logistic regression model to account for potential confounders and stratification factors. Continuous outcomes will be analysed using general linear models. Correlation between repeated measures per patient will be appropriately accounted for in the linear models where applicable. Binary data will be analysed using logistic regression where there are no repeated data per patient. Repeated binary data will be analysed using generalised estimating equations. Stratification factors and chance baseline imbalances following randomisation will be explored for the primary and secondary outcomes.

Analysis will follow intention-to-treat principles with patients analysed according to the surgery allocated by randomisation and irrespective of surgery received, subsequent management or events [26]. Every effort will be made to retain and include all patients who receive surgery as part of the trial.

A prospective economic evaluation is integrated into the trial design and applies an NHS perspective to the inclusion of costs. Mechanisms of missingness within the data will be explored, and multiple imputation methods will be applied to impute missing data and minimise bias. Imputation sets will be used in bivariate analysis of costs and QALYS to generate incremental cost per QALY estimates and credible intervals [27–29]. It is anticipated that incremental costs and benefits will be captured within the trial, although extrapolated economic modelling will be considered if appropriate. Findings will be presented on the incremental cost-effectiveness ratio (ICER) plane and using cost-effectiveness acceptability curves (CEACs).

### Governance

The trial is overseen by a TSC, which includes an independent chair and two other independent members (one of whom is a patient). In addition, the trial has a DMC, which meets 6 monthly, and oversees all ethical and safety issues in accordance with a study-specific DAMOCLES charter [30] for DMCs. All members are independent of the study team, although the Trial Manager, Chief Investigator and some other members of the Trial Management Group (TMG) attend the open sessions in order to inform the DMC of trial progress.

### Reporting of adverse events

AEs and serious adverse events (SAEs) are recorded and reported from the time of index surgery until completion or withdrawal. SAEs are reported within 24 h of the research team becoming aware of the event to the Sponsor. Where required, these events undergo expedited reporting to the Research Ethics Committee. All AEs are assessed for severity, causality, expectedness and seriousness by an Investigator; all are reviewed by the DMC.

### Dissemination

On-going patient and public involvement informs appropriate methods of dissemination to patients. Feedback will be given to national surgical leads via the Society for Cardiothoracic Surgeons in Great Britain and Ireland, maximising the exposure of findings to cardiac surgeons diagnosing and treating patients requiring AVR.

Findings will be presented at national specialist meetings to raise awareness. We will engage directly with surgeons and cardiothoracic units around the country to share results. Data will be presented at the annual meeting of the Society for Cardiothoracic Surgeons in Great Britain and Ireland, and we anticipate this to be the main forum for disseminating findings from this study.

## Discussion

This is the first trial to examine aortic valve replacement via manubrium-limited ministernotomy versus conventional sternotomy when comparing red blood cell transfusion rates following surgery. MAVRIC will determine if manubrium-limited ministernotomy should be adopted as best practice for patients requiring AVR surgery.

It was not possible to blind clinicians to the surgical procedure provided, although transfusion decisions are protocol-driven and should not be procedure-related. The inclusion of patient blinding was in response to a funding panel recommendation. It has been possible to implement this through the use of opaque dressing, which means that patient-reported pain scores at 2 days will be blinded. We will assess the effectiveness of blinding by inviting patients to indicate which treatment they think they have received before removing the dressings.

Surgical trials present significant challenges. The strengths of this trial include a rigorous research design, standardised surgery performed by experienced consultant cardiothoracic surgeons, an agreed anaesthetic regimen, patient blinding and consultant-led patient recruitment. Each discipline within the Cardiothoracic Division at JCUH is supporting and collaborating with the Chief Investigator. MAVRIC will demonstrate that complex surgical trials can be delivered to exemplary standards and provide the community with the knowledge required to inform future care for patients requiring aortic valve replacement surgery.

### Protocol version

This is approved version 5 of the trial protocol.

### Trial status

The trial began recruiting in March 2014; the trial is due to report in 2017.

## Additional file

Additional file 1: SPIRIT 2013 checklist. (DOC 121 kb)

## Abbreviations

ACT: activated clotting time
AE: adverse event
APTT: activated partial thromboplastin time
APTTR: activated partial thromboplastin time ratio
AVR: Aortic valve replacement
CEACs: cost-effectiveness acceptability curves
CICU: Cardiac Intensive Care Unit
CPB: cardiopulmonary bypass
DCTU: Durham Clinical Trials Unit
DMC: Data Monitoring Committee
e-CRFs: electronic Case Report Forms
FEV1: forced expiratory volume
FFP: Fresh frozen plasma
FVC: forced vital capacity
GCP: Good Clinical Practice
GP: General Practitioner
Hb: haemoglobin
ICER: incremental cost-effectiveness ratio
ICH: International Conference on Harmonisation
INR: International normalized ratio
JCUH: The James Cook University Hospital
NHS: National Health Service
NIHR: National Institute for Health Research
PT: prothrombin time
PTR: prothrombin time ratio
QALY: quality-adjusted life year
RBC: Red Blood Cell
RfPB: Research for Patient Benefit
SAE: serious adverse events
SIR: systemic inflammatory response
SPIRIT: Standard Protocol Items: Recommendations for Interventional Trials
TEG: Thromboelastography
TMG: Trial Management Group
TOE: transoesophageal echocardiogram
TSC: Trial Steering Committee
TXA: tranexamic acid
